# Fused Audio Instance and Representation for Respiratory Disease Detection

**DOI:** 10.3390/s24196176

**Published:** 2024-09-24

**Authors:** Tuan Truong, Matthias Lenga, Antoine Serrurier, Sadegh Mohammadi

**Affiliations:** 1 Bayer AG, 13353 Berlin, Germany; matthias.lenga@bayer.com (M.L.); sadegh.mohammadi@bayer.com (S.M.); 2 Clinic for Phoniatrics, Pedaudiology and Communication Disorders, University Hospital of RWTH Aachen, 52074 Aachen, Germany; aserrurier@ukaachen.de

**Keywords:** audio, waveform, spectrogram, multi-instance learning, deep learning, classification, respiratory disease, COVID-19

## Abstract

Audio-based classification techniques for body sounds have long been studied to aid in the diagnosis of respiratory diseases. While most research is centered on the use of coughs as the main acoustic biomarker, other body sounds also have the potential to detect respiratory diseases. Recent studies on the coronavirus disease 2019 (COVID-19) have suggested that breath and speech sounds, in addition to cough, correlate with the disease. Our study proposes fused audio instance and representation (FAIR) as a method for respiratory disease detection. FAIR relies on constructing a joint feature vector from various body sounds represented in waveform and spectrogram form. We conduct experiments on the use case of COVID-19 detection by combining waveform and spectrogram representation of body sounds. Our findings show that the use of self-attention to combine extracted features from cough, breath, and speech sounds leads to the best performance with an area under the receiver operating characteristic curve (AUC) score of 0.8658, a sensitivity of 0.8057, and a specificity of 0.7958. Compared to models trained solely on spectrograms or waveforms, the use of both representations results in an improved AUC score, demonstrating that combining spectrogram and waveform representation helps to enrich the extracted features and outperforms the models that use only one representation. While this study focuses on COVID-19, FAIR’s flexibility allows it to combine various multi-modal and multi-instance features in many other diagnostic applications, potentially leading to more accurate diagnoses across a wider range of diseases.

## 1. Introduction

The human body produces numerous sounds that indicate its state of health. A slight change in an organ’s physical state can impact its operation, leading to irregular sound patterns. Snoring, for example, is a common sound produced by upper airway obstruction during sleep. While snoring alone is generally not considered pathological, if coupled with breathing pauses, it can signal obstructive sleep apnea [1]. More generally, body sounds can be used extensively to support diagnostic decisions. In particular, auscultation is a common technique used by clinicians to listen to internal sounds of the body with a stethoscope. Abnormal patterns in organs such as the heart, the lungs, and the gastrointestinal system can be detected using this method. In respiratory diseases such as pneumonia, auscultation can be performed to look for crackles or tubular breath sounds, an indication of pulmonary consolidation [2]. Hence, body sound analysis is part of computer-aided diagnostic applications such as in respiratory diseases [3,4,5,6], Parkinson’s disease [7], and sleep apnea [8]. Although detecting irregular internal sounds might be insufficient for a definitive diagnosis, it serves as an important indicator that can be combined with other confirmatory clinical tests from different diagnostic tools to reach a conclusive diagnostic decision.

In this study, we explore an audio-based approach for screening respiratory diseases, focusing on coronavirus disease 2019 (COVID-19). This disease, caused by SARS-CoV-2, infects the respiratory tract [9] and can be difficult to differentiate from other respiratory illnesses. Viral testing through nucleic acid tests such as polymerase chain reaction (PCR) is a gold standard but takes several hours or even days to deliver results. Additionally, PCR testing requires specialized personnel and equipment that may not be available in low-income or remote areas. An alternative test, known as the antigen test, can retrieve results in less than 30 minutes by identifying viral proteins with specific antibodies. It is a viable option for mass testing but is less sensitive. The authors in [10] set a minimum accepted sensitivity of 75% for rapid antigen tests and find that many test kits in the market miss this threshold. Since SARS-CoV-2 infects mainly the respiratory system, it can induce changes in body sounds such as voice and breath. This includes dysphonia, breath abnormalities, and coughs. Several studies suggest that these changes correlate to COVID-19. For example, a study by Huang et al. [11] finds abnormal breathing sounds in all COVID-19 patients, including crackles, asymmetrical vocal resonance, and indistinguishable murmurs. Another study [12] validates the link between abnormal vocal fold oscillations and COVID-19, explaining voice changes and speaking difficulties. Respiratory and vocal sounds, therefore, have the potential to be used as a standalone test or to be combined with antigen tests for COVID-19 detection.

Screening COVID-19 using respiratory and vocal sounds offers several advantages. Firstly, with limited PCR testing capacities, sound-based screening combined with antigen tests can help prioritize who is eligible for PCR tests. Allowing anyone with flu-like symptoms to order a PCR test would swiftly overwhelm the testing capacity. Only individuals flagged by respiratory and vocal sound screening could proceed with confirmatory PCR tests. Sound-based screening can rapidly identify suspect cases without asking them to quarantine while waiting for PCR results. Secondly, like antigen tests, sound-based screening is fast, affordable, convenient, and can be conducted without medical professionals. The cost of running respiratory and vocal sound screening can even be lower than that of antigen tests because it can be installed as software or a mobile application on any device, utilizing existing device microphones and avoiding the need for additional support kits. Users can record, analyze, and monitor their status unlimited times on their devices. This is particularly useful in regions or countries where testing capacities are scarce, inaccessible, or expensive. Lastly, compared to antigen tests, sound-based screening generates no medical waste because no physical products are manufactured, which alleviates the environmental burden.

Respiratory and vocal sounds hold great promise for non-invasive COVID-19 screening. However, a fully developed screening system is not yet available. Current research on COVID-19 detection considering multiple body sounds often focuses on individual sounds, neglecting their interaction [13,14]. COVID-19 may manifest in different body sounds or combinations of them, varying across individuals. One or more body sounds may be affected, while the others remain intact. It is thus sensible not to rely on a single one but rather on a combination of several body sounds. We propose combining the most indicative body sounds for COVID-19 using fusion rules within the detection algorithm. We hypothesize that the cough, breath, and speech sounds contain biomarkers for COVID-19 and can be combined using an appropriate fusion rule to maximize the chances of correct detection. To this end, we propose self-attention as a fusion rule to combine features extracted from cough, breath, and speech sounds. Mainly, we use waveforms and spectrograms as the inputs to our model. A waveform represents an audio signal in the time domain, whereas a spectrogram is a representation in the time-frequency domain. Our main contributions in this work are summarized as follows:

- We demonstrate that cough, breath, and speech sounds can be leveraged to detect COVID-19 in a multi-instance audio classification approach based on self-attention fusion. Our experimental results indicate that combining multiple audio instances exceeds the performance of single-instance baselines.
- We experimentally show that an audio-based classification approach can benefit from combining waveform and spectrogram representations of input signals. In other words, inputting the time- and frequency-domain dual representations into the network allows for a richer latent feature space, ultimately improving the overall classification performance.
- We integrate the above contributions into the FAIR approach, a method that combines multiple instances of body sound in waveform and spectrogram representations to classify negative and positive COVID-19 individuals. The FAIR approach is a general concept that can be applied to other sound classification tasks such as those related to other respiratory diseases.

## 2. Related Work

Body sound analysis for pulmonary disorders has long been studied with diagnostic applications in tuberculosis [5,15], pneumonia [3], chronic obstructive pulmonary disease (COPD) [6,16,17], asthma [16], croup, and pertussis [18]. Additionally, there are studies on the classification of specific lung sounds, such as wheezes and crackles [19,20]. Datasets in these studies are relatively small, ranging from tens to a few hundred subjects, and often not publicly available [3,5,15,19]. The authors commonly rely on handcrafted audio features, such as mel-frequency cepstral coefficients (MFCC), log spectral energies, zero-crossing rate (ZCR), and kurtosis. Some works leverage both handcrafted features and deep learning [17,18] and study the model’s explainability [21]. While most studies achieve an overall area under the receiver operating characteristic curve (AUC), sensitivity, and specificity over 0.9, the limited training data and reliance on handcrafted features may present challenges for the generalization of proposed approaches. Recent and detailed reviews of disease classification from cough and respiratory sounds can be found in the work of Serrurier et al. [22] and Xie et al. [23].

The COVID-19 pandemic has fueled significant research growth and the development of new techniques and datasets specifically focused on COVID-19 detection. Large collections of COVID-19 sounds have been created through crowdsourcing. Voluntary participants submit recordings of their body sounds to a mobile app or website and provide metadata such as their COVID-19 status and comorbidity. Such large datasets enable researchers to develop COVID-19 detection algorithms. To our knowledge, the largest crowdsourcing datasets are COUGHVID [24], Coswara [25], and COVID-19 Sounds [26]. COUGHVID comprises more than 20,000 cough recordings, while Coswara and COVID-19 Sounds consist of cough, breath, and vocal sounds from more than 2000 and 30,000 participants, respectively. In terms of technical development, some studies utilize traditional machine learning approaches with the handcrafted features mentioned earlier [13,27,28,29,30]. On the other hand, several studies adopt deep learning approaches by training a convolutional neural network (CNN) on spectrograms or waveforms instead of handcrafted features. Rao et al. [31] presented a VGG13 network [32] that utilizes spectrograms as the input with a combined cross-entropy and focal loss. Their approach achieved an AUC of 0.78 on the COUGHVID dataset. Early works that combine different respiratory sounds and demonstrate improved classification performance are Xia et al. [33] and Wall et al. [34]. Xia et al. [33] analyzed concatenated features of cough, breath, and speech sounds in a simple VGG-ish model. The study introduced the combination of features from various body sounds to improve classification performance, achieving an AUC of 0.75 and a sensitivity and specificity of 0.70. Wall et al. [34] put forward an ensemble approach by combining four deep neural networks with attention mechanism. The ensemble model, trained separately on respiratory, speech, and coughing audio from the ICBHI and Coswara datasets, achieved overall performance for the base and ensemble model with ICBHI scores between 0.920 and 0.9766. While the ensemble approach is shown to benefit the performance of the classification task, it does not allow studying the interaction among different respiratory and vocal sounds as the models are trained separately for each sound type.

In our study, we investigate the former approach, which seeks to combine body sound instances. Unlike research works that usually study each body sound independently [14] or combine them by voting on prediction scores [13,34], we explore fusion rules that combine them at the feature level. In other words, we train a network to learn a joint feature vector that incorporates all respiratory and vocal sounds. The joint feature vector is optimized to implicitly reflect the relative importance of each body sound for the final prediction. Although our work shares similarities with Xie et al. [33], we investigate a more complex fusion rule than simply concatenating features. We use self-attention [35], which captures the dependencies among body sounds into a joint feature vector. Self-attention is not only used as a layer in the transformer architecture but also for feature aggregation [36]. This is considered late fusion, as opposed to early fusion, as in the work of Wanasinghe et al. [37], where the authors concatenate the features channel-wise to form an input for the classification model. In addition, instead of using handcrafted audio features, we train our model directly on waveform and spectrogram representations, creating more robust features compared to previous methods. We report an average performance of the models obtained from cross-validation on a split test set in Section 3. It is important to note that the Coswara dataset does not have a single and universally used test set, and the data size was growing at the time we conducted our experiment.

## 3. Methods

**Figure 1:**
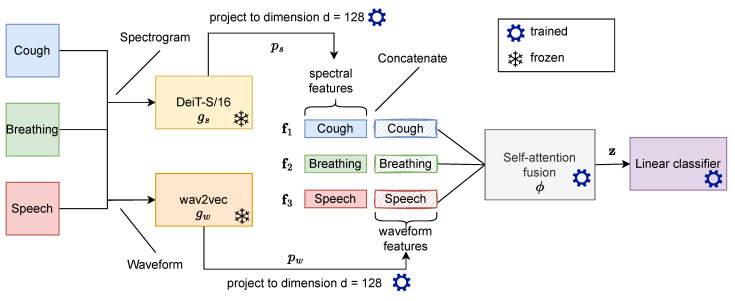
An overview of the FAIR approach. FAIR is an end-to-end approach consisting of two stages: feature extraction and feature fusion. In the first stage, the pretrained wav2vec and DeiT-S/16 extract waveform and spectrogram features from body sounds (here c=3), which are projected to the embedding of dimension d=128. In the second stage, the embeddings of multiple instances and representations are fused into a compact feature vector using self-attention. The resulting joint feature vector is used by the classifier, which is a two-layer MLP that outputs the probability of COVID-19 infection.

Fused audio instance and representation (FAIR) for COVID-19 detection is an end-to-end approach that consists of feature extractors for waveform and spectrogram representations, an attention-based fusion unit, and a classifier, as depicted in Figure 1.

For ease of exposition, assume that the system should consider *c* different input (body) sounds (for example, see Section 4.1). Each input sound is represented by a fixed-length *waveform* vector and the associated *spectrogram* representation. The fixed-length waveform vectors $x_{1},...,x_{c}\in R^{l}$ are obtained by resampling and optionally padding the original input audio signals. The associated spectrograms $x_{1+c},...,x_{2c}\in R^{m\times n}$ are constructed by transforming the waveform representation with the discrete short-time Fourier transform [38], where *m* is the number of time frames and *n* is the number of frequency bins. In our experiments, we use the mel-spectrogram, which is the logarithmic transformation of the frequency in hertz to mel scale given by the equation

(1) $$f_{Mel}=1127\ln\left(1+\frac{f_{Hz}}{700}\right).$$

In order to obtain a representative joint feature vector for all *c* input body sounds across waveform and spectrogram inputs, we utilize two pre-trained neural networks $g_{w},g_{s}$ followed by linear layers $p_{w},p_{s}$ to project the waveform and spectrogram into a common feature space, respectively. The concatenated projections $f_{1},...,f_{c}$ where

(2) $$f_{k}=\left[p_{w}\circ g_{w}\left(x_{k}\right),p_{s}\circ g_{s}\left(x_{k+c}\right)\right]\in R^{2d}$$

are then fused using an attention-based fusion unit $\phi :R^{c\times 2d}\to R^{d}$ to obtain a reduced joint feature representation

(3) $$z=\phi \left(f_{1},f_{2},...,f_{c}\right)\in R^{d}.$$

Figure 1 shows an overview of the FAIR approach and the main components along the pipeline. The feature extractors and the attention-based fusion unit are instrumental components in our proposed approach and are further detailed in the next sections.

### 3.1. Feature Extractors

Feature extractors are neural networks responsible for learning representative features for each body sound. As the input consists of waveform and spectrogram, two neural networks $g_{w}$ and $g_{s}$ are trained in parallel to handle both representations of the audio data. All waveform inputs are transformed via $g_{w}$ and the corresponding spectrograms are transformed via $g_{s}$ to latent representations. We choose for $g_{w}$ a pretrained wav2vec [39] network, and for $g_{s}$, we choose a DeiT-S/16, a vision transformer (ViT) model [40], as backbone. DeiT-S/16 and wav2vec are transformer-based models and achieve state-of-the-art results in language and vision models.

**Figure 2:**
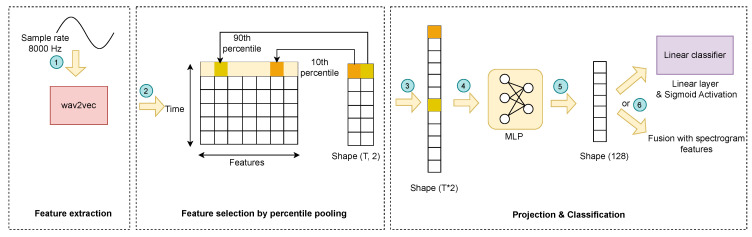
Wav2vec-based extraction of waveform features. Step 1: 16 kHz sampled audio is fed into the pretrained wav2vec model to extract features. Step 2: wav2vec outputs a feature vector per every 25 ms of the audio, resulting in a t×d matrix, where *t* is the total time indices and *d* is the dimension of the feature vector. We select in each feature vector the element at the 10th and 90th percentile. Step 3: The new feature matrix is flattened into a single vector. Step 4: An MLP layer receives the feature vector. Step 5: The MLP layer projects it into a fixed dimension of 128. Step 6: The resulting feature vector is fed into a linear classifier or can be fused with other features (FAIR method; see Figure 1) before entering the linear classifier. The linear classifier is a linear layer with one neuron followed by sigmoid activation that outputs the predicted probability of a COVID-19 infection.

The wav2vec network [39], originally developed for speech-to-text translation tasks, comprises both convolutional and self-attention layers. It is pretrained on a large audio corpus in an unsupervised fashion. Therefore, we take advantage of the pretrained wav2vec features and design a fine-tuning unit to effectively utilize them in our COVID-19 detection task. As shown in Figure 2, the recording is first resampled to 16,000 Hz. We then extract features every 25 ms using the pretrained wav2vec model without changing its weights. As features are only extracted for every 25 ms time frame, we use percentile pooling to aggregate features across all frames. For each feature along the time axis, we select the values at the 10th and 90th percentile. This is considered a robust alternative to the min and max pooling of feature vectors because the min and max values might output outliers due to the background noise in the recordings. The 10th and 90th percentiles, therefore, represent the bottom and top 10% of feature values while excluding outliers. Our tuning experiment also shows that percentile pooling results in superior performance compared to just average or median pooling. After this step, we flatten the resulting feature matrix and feed it into a MLP layer to reduce the dimensions of the feature embedding to 128.

The DeiT-S/16 architecture is a variant of ViT introduced by Touvron et al. [41] as part of the data-efficient image transformers (DeiT). It has the exact architecture of the original ViT [40] and differs only in the training strategy. The utilized model is categorized into the small (S) transformer family, where the projected embedding dimension through self-attention blocks is 384. It consists of 12 multi-headed self-attention (MSA) layers [35], each consisting of six heads. The resolution of each patch in the attention layer is 16 × 16 pixels. We modify the last dense layer of DeiT-S/16 to be an identity unit to extract features from the previous layers. In all our experiments, we use a pretrained DeiT-S/16 on the ImageNet dataset and fine-tune it on our target dataset. Finally, we projected the output to a 128-dimensional feature vector similar to wav2vec.

### 3.2. Fusion Unit and Classifier

The fusion unit ϕ combines the projected joint embeddings $f_{1},...,f_{c}$ as defined in (2) into a single vector z by using an MSA layer [35] and an MLP *h*:

(4) $$z=\phi \left(f_{1},...,f_{c}\right)=h\left(\mathrm{MSA}\left(f_{1},...,f_{c}\right)\right).$$

Self-attention is originally developed for language models. In language models, a sequence consists of many tokens (e.g., words) that the model processes to capture the overall meaning (i.e., global information). Similarly, in our case, the tokens are feature vectors representing different sound instances. While memorizing long sequences can be challenging, and models may struggle to retain information from the beginning of the sequence, self-attention addresses this by dynamically creating a new set of features by linearly combining the original feature vectors. In detail, the output of MSA for a given input feature sequence $f_{1},...,f_{c}$ is a new set of feature vectors $f_{1}^{\prime },...,f_{c}^{\prime }$, where each $f_{k}^{\prime }$ is obtained as a weighted combination of the original feature vectors:

(5) $$\left[f_{1}^{\prime },...,f_{c}^{\prime }\right]=\mathrm{softmax}\left(\frac{{\mathrm{QK}}^{⊤}}{\sqrt{d}}\right)V.$$

Here, Q, K, and V are linear projections of $\left[f_{1},...,f_{c}\right]$ with learnable matrices $W_{q}$, $W_{k}$, and $W_{v}$. The output of the softmax operation corresponds to the attention matrix related to the input features (cf. [35]).

Next, all feature vectors $f_{1}^{\prime },...,f_{c}^{\prime }$ are concatenated and projected using an MLP *h*, first to 256 dimensions and finally to a 128-dimensional feature vector z. For the classifier, we selected a linear layer with a single output neuron followed by a sigmoid activation function. It maps the fused representation z to the predicted class probability score.

## 4. Experiment

### 4.1. Dataset

**Figure 3:**
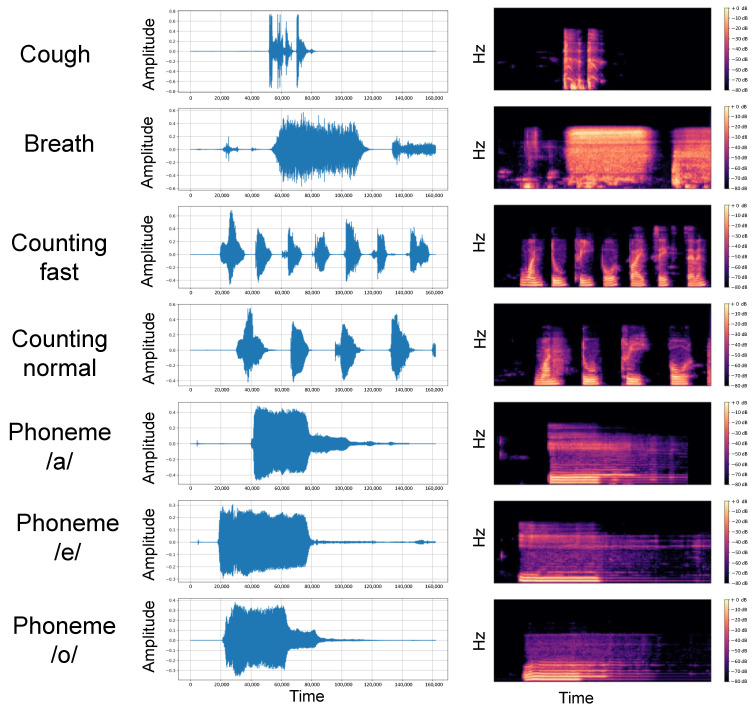
Visualization of the data in our study. We plot the waveform and associated mel spectrogram of 5 body sounds, namely cough-heavy, breath-deep, counting-fast, counting-normal, and phonemes /a/, /e/, and /o/. We do not use the shallow cough and breath due to the high noise level. The recordings are resampled to 44,100 Hz and visualized with the first 4 s.

Coswara is a crowdsourcing project to build an audio corpus from COVID-19-negative and -positive individuals. The dataset is publicly available to enable research on diagnostic tools for respiratory diseases, particularly COVID-19. The dataset is published in the work of Bhattacharya et al. [42] and publicly available at https://github.com/iiscleap/Coswara-Data (accessed on 1 September 2021). Approval of data collection was issued by the Institutional Human Ethics Committee at the Indian Institute of Science, Bangalore. Informed consent was obtained from all participants who uploaded the recordings. The collected data were anonymized and de-identified by the dataset’s provider. The audio recordings were collected between April 2020 and February 2022. Data collection occurs through a web interface where users are prompted to provide their metadata and recordings using a device microphone. The metadata cover age, sex, location, and COVID-19 status. Users are then instructed to submit nine audio recordings of (heavy and shallow) cough, (deep and shallow) breath, (fast and slow) counting from 1 to 20, and uttering the phonemes /a/, /e/, and /o/. The COVID-19 status must be selected from the categories negative, positive with or without symptoms, recovered, and no identified respiratory disease. There is no restriction on the duration of the recordings, so users can decide when they want to start and stop recording. Figure 3 visualizes the waveforms and spectrograms of a participant in the Coswara dataset. We accessed the database when it was still in the last collection stage. The recordings used in our study have timestamps between 14 March 2020 and 14 July 2021. All methods in our study were carried out in accordance with relevant guidelines and regulations.

### 4.2. Data Preprocessing and Augmentation

Regarding data preprocessing, we first removed the leading and trailing silence. We observed that long recordings (>20 s) mainly contain silence, and the duration at which people cough, breathe, or speak lasts only 3–10 s. Next, we removed corrupted files, which are those that contain no sound or noise or a different sound type than the one reported in the label. The recordings whose duration is less than 1 s were eliminated because they do not contain any detected sound. Then, similar to the approach of [33], we used a pretrained model called YAMNet, trained on a massive dataset of YouTube audio events (including cough, speech, and breath), to systematically remove recordings where the detected sound does not match the provided label. In addition, we excluded shallow cough and breath based on the provided labels in our experiments due to low quality and high misdetection rate as noise. After preprocessing, 735 patients were removed, leaving 1359 participants for analysis, with 223 COVID-19 positive and 1136 COVID-19 negative. Each participant has exactly 7 recordings, which amounts to 9513 recordings used in our experiments. Table 1 provides statistics on the audio length. The participants were split into six folds for training and testing, and details are provided in Section 4.4.

We used Torchaudio (version 0.9.1) for audio processing and normalization. The values of loaded audio are automatically normalized between −1 and 1. Recordings were resampled to two rates: 44,100 Hz (DeiT-S/16) and 16,000 Hz (wav2vec). We found that the first 4 s of each recording yield the best performance after tuning with different lengths. For spectrogram transformation, we took the mel-spectrogram with 128 mel filterbanks operating in 1025 frequency bins, i.e., FFT size of 2048, window size of 2048, and hop size of 1024. We performed data augmentation on the fly during training. This means we randomly selected a continuous 4 s interval from the first 5 s of each recording, introducing a slight variation. However, during evaluation, we consistently selected the first 4 s. We investigated many audio augmentation techniques such as pitch shift, time stretch, or masking, but only amplitude scaling, time, and frequency masking improve performance. Amplitude scaling randomly injects an amplitude gain between 0.9 and 1.3 on the waveform. Amplitude scaling is always performed after normalization and before spectrogram transformation. Additionally, we applied random time and frequency masking to the spectrogram, where a block of data is set to zero for a duration of 10 units (time or frequency steps).

### 4.3. Baseline and Benchmark Experiments

We compare the models developed with a single body sound instance, the baseline (BA), with multiple combinations of body sounds, the benchmark (BE). Table 2 shows an overview of the baseline and benchmark experiments. In baseline experiments, we train seven models, each using only a single body sound (heavy cough, deep breath, fast and normal counting, and the utterance of the phonemes /a/, /e/, and /o/). In the benchmark experiments, we group counting and utterance of the three vowels as a single instance, thereafter speech. We investigate the following combinations: (1) speech, (2) cough and breath, (3) cough and speech, (4) breath and speech, and (5) cough, breath, and speech. In both the baseline and benchmark experiments, we use either waveforms or spectrograms in separate experiments. The last experiment (BE3) is our FAIR model, which utilizes both waveform and spectrogram. The input to DeiT-S/16 [41] is a spectrogram image of size 128 × 173 calculated from a 4 s audio clip sampled at 44,100 Hz. The waveform input to wav2vec has a sample rate of 16,000 Hz to be compatible with its pretraining, resulting in a vector length of 64,000 for a 4 s clip.

### 4.4. Cross-Validation

A set of 226 subjects (191 COVID-19 negative and 35 positive), thereafter the test fold, is randomly selected from our data to serve as a fixed test set for all experiments. The remaining 1133 subjects are used as training and validation in a five-fold cross-validation scheme as follows: the subjects are split into five folds of similar size (see Table 3), four folds are used for training, and the remaining fold is used for validation in a rotating process so that each subject is used exactly once as the validation fold. It provides five different models. Each of them is tested on the fixed test fold, and the average of the results is reported.

### 4.5. Hyperparameters

Table 4 shows the complete hyperparameter settings in our experiments. Most hyperparameters are identical across architectures, representations, or fusion rules. For example, we train all models for 30 epochs without early stopping, and the best checkpoint is saved based on the best AUC obtained in the validation fold. The loss function that we use is binary cross-entropy (BCE), and we optimize this loss with AdamW (Adam with weight decay) [43], which is often used with transformer-based architecture [35]. We fix a base learning rate of 0.0001 for all experiments and adjust the learning rate scheduler and weight decay conditional on the architecture or fusion rules. The weight decay factor is set between 0.1 and 0.001. These hyperparameters are experimentally chosen with cross-validation.

### 4.6. Training

All models in our experiments are trained end-to-end, meaning all components (feature extractors’ projection layers, attention-based fusion unit, and linear classifier) are trained simultaneously. The pretrained wav2vec and DeiT-S/16 are frozen, and only the added projecting layers are updated during training. The number of trainable parameters for the FAIR approach can be found in Table A8 in Appendix A. To address the class imbalance in the dataset, we employ two techniques: weighted loss and batch oversampling. Weighted loss assigns a higher penalty for misclassifying the minority class (COVID-19 positive), encouraging the model to focus on learning from these rarer examples. Batch oversampling ensures an equal representation of positive and negative classes within each training batch, further mitigating bias towards the majority class.

### 4.7. Evaluation

Our primary metric for model selection is AUC. During training, we save the checkpoint with the highest performance based on AUC. During validation, we use the ROC curve to compute the optimal threshold, which is the threshold resulting in the maximum sum of sensitivity and specificity, and take this threshold to compute other metrics such as sensitivity and specificity in the test set. We report the AUC scores in the main paper and provide the sensitivity, specificity, and area under the precision–recall curve (AUPRC) in Appendix A. For statistical testing, our samples are dependent and not normally distributed by the Kolmogorov–Smirnov test. Therefore, we opt for the one-tailed Wilcoxon signed-rank test with n = 10. This means we repeat the five-fold cross-validation twice (with different random seeds) and use the Wilcoxon test with a significance level of alpha 0.05 to compare the performance of different models.

## 5. Results

### 5.1. Baseline Results

Table 5 shows the performance of the models trained on a single body sound instance. The input to the model is either a waveform (BA1) or a spectrogram (BA2) of a single body sound. The results reveal that the models trained on spectrograms perform substantially better than those trained on waveforms. The average AUC scores for DeiT-S/16 (BA2) and wav2vec (BA1) are 0.7549 and 0.6127. The performance of different body sounds across architectures and representations does not establish a consistent pattern. For example, using only cough sounds leads to the highest AUC score in DeiT-S/16, but a lower score in wav2vec. There appears to be a countertrend between DeiT-S/16 and wav2vec. For example, the counting sound achieves better results than the fast counting sound in DeiT-S/16 but worse in wav2vec. Similarly, the utterance of /o/ outperforms other vowels in DeiT-S/16 but performs poorly in wav2vec.

### 5.2. Benchmark Results

Table 6 presents the results comparing the FAIR model (BE3) to the DeiT-S/16 (BE2) and wav2vec (BE1) models across various body sound combinations using self-attention fusion. A one-tailed Wilcoxon signed-rank test statistically evaluates the performance of BE3 to BE1, BE2, and all BA experiments. The FAIR approach generally outperforms models trained on a single representation. The sole exception is the cough-breath combination, where the *p*-value exceeds 0.05 for all individual body sounds except fast counting. Within benchmarking experiments, the FAIR approach demonstrates statistically significant improvement (*p* < 0.05) compared to using a single feature extractor, with the exceptions of cough-breath and cough-speech combinations, which exhibit *p*-values exceeding 0.05. The average AUC score of FAIR is 0.8316, which is 0.0227 more than DeiT-S/16 and 0.0847 more than wav2vec. FAIR achieves the highest AUC scores in all combinations of body sound, with the only exception in the cough-breath combination, which will be discussed in the next section. The cough-breath combination results in the lowest AUC score in all alternatives in terms of the body sound combination. The largest combination, cough-breath-speech, gives the best results in FAIR and wav2vec but is behind the cough-speech combination in DeiT-S/16 by a margin of AUC 0.007. FAIR achieves the highest AUC score of 0.8658 with the combination of cough, breath, and speech. This score is 0.0343 and 0.0941, higher than the best scores produced by DeiT-S/16 and wav2vec. The results of the FAIR models find clear support for the use of dual audio representation along with body sound fusion.

## 6. Discussion

**Figure 4:**
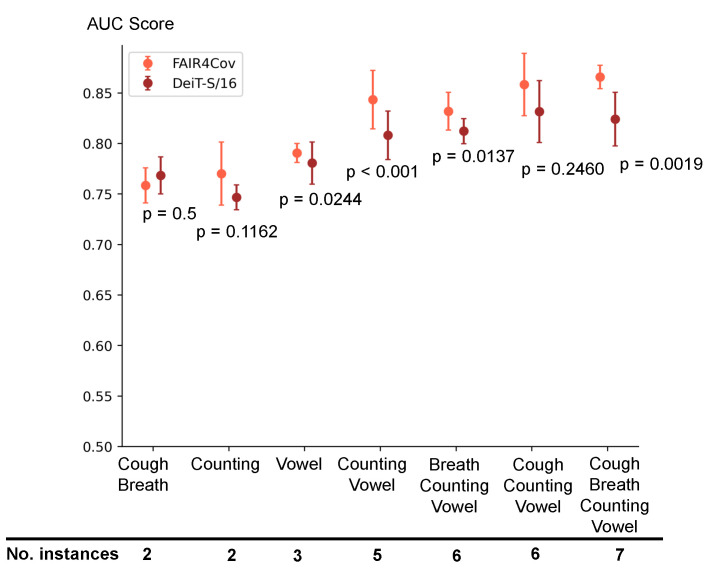
Comparison of the DeiT-S/16 model (spectrogram features) and FAIR (waveform and spectrogram features). The x-axis shows the combination with the number of instances in ascending order of quantity. Additional results can be found in Table A2 and Table A4 in Appendix A.

As can be seen in Table 6, the AUC scores vary among body sound combinations, making it unclear which combination is best. Therefore, it is valid to doubt whether a preferable combination of body sounds leads to the best predictive outcome. However, neither our results nor the literature provide a conclusive answer. We suggest that performance is correlated with the number of body sounds in a combination. To illustrate, we compare the performance of the model trained with (1) a single body sound instance and (2) a combination of body sounds. In training models with a single body sound instance as input (Section 5.1), no single body sound consistently outperforms the others. The best-performing sound depends on the architecture or audio representation used. For instance, the cough sound performs well with DeiT-S/16 (BA2) but not with wav2vec (BA1). Similarly, in our ablation study, replacing DeiT-S/16 with ResNet50 yields similar results (Table A3 in Appendix A). These subtle differences among body sounds may be due to the stochasticity or the feature extractor settings, indicating that no body sound is significantly better than the others as input to our model. Regarding the combinations of body sounds (Section 5.2), we observe that the combination of cough and breath consistently yields the lowest AUC scores for all models. This combination involves only two body sound instances, while all others include at least five instances. This observation suggests that the performance is likely to correlate with the number of instances of body sound. To support this, we conduct additional experiments in a similar setting to benchmark experiments with the following combinations: counting (incl. fast and normal counting) and vowel (incl. utterance of /a/, /e/, and /o/). Figure 4 shows that counting and cough-breath combinations perform similarly, while the three vowel utterances outperform the two-instance combinations by 0.03–0.04 AUC. This supports a correlation between performance and the number of body sounds.

We analyze the effect of the dual representation of the spectrogram and waveform in the absence of body sound fusion by conducting an ablation study similar to the FAIR framework but with the input of a single body sound. As there are no rules for body sound fusion, the features extracted from two representations are concatenated, flattened, and then projected onto a 128-dimensional vector by an MLP layer. Similar to the baseline experiment, we present the AUC scores of seven models trained on seven body sound instances in Table 7. Overall, the average AUC scores are on par with those of the DeiT-S/16 model (BA2) in Table 5. Breath and counting sounds achieve the highest AUC score, whereas the utterance of vowels /e/ and /o/ leads to the lowest performance. The benefit of joint features from dual representation is not observed because the change in the individual AUC scores of each body sound does not follow any pattern. Compared to the DeiT-S/16 results in Table 5, except for cough, the difference in performance is subtle. The result suggests that the waveform representation contributes little to the final classifier. The performance is indeed strongly influenced by the powerful DeiT-S/16 in the spectrogram representation, which eclipses the features obtained from the waveform. Therefore, we conclude that using dual representation in the absence of body sound fusion does not improve any performance. However, when the dual representation is used for body sound fusion, the extra information from multiple body sounds is picked up by the fusion unit and enriches the joint extracted feature. The fusion unit is able to amplify the aggregated information due to the self-attention mechanism. One of the interesting properties of self-attention is scaling, which is discussed in the work of Dosovitskiy et al. [40]. The authors note that the performance of the transformer-based model could be scaled up in response to an increase in the resolution of patches or number of blocks. This contrasts with convolutional networks, in which accuracy can reach saturation at a certain level of complexity. This scaling property explains why adding more body sounds leads to a steady increase in AUC scores. Adding more body sounds means adding more tokens and establishing stronger dependencies among them. When only two or three instances of body sound are adopted, the effect of body sound fusion is less significant. Figure 4 shows the AUC scores of the FAIR and DeiT-S/16 models on the different combinations of body sounds sorted in ascending order of instances. Combinations with less than or equal to three instances (i.e., cough-breath, fast and normal counting, /a-e-o/ vowel utterance) achieve AUC scores in the range of 0.75–0.79, which is on par or slightly better than the performance of models on a single instance (Table 7). This happens because the number of instances is insufficient to establish long-range dependencies. As more body sounds are added, these dependencies are captured, and the performance of models with fusion units starts to improve substantially. We observe a similar effect when replacing the fusion unit of FAIR by attention-weighted pooling (see Table A7 in Appendix A). When the number of body sounds in the combination is less than three, both attention-based fusion units have comparable performance. However, the gap is significant as more instances are combined. In addition, the joint feature vector embeds more information when a dual representation is adopted. When the number of instances in the combination is small, i.e., less than three, the gain due to the dual representation is not noticeable. However, starting from five instances, the gap between FAIR and DeiT-S/16 becomes wider in favor of FAIR. We attribute this gain to the resonance of extra information given by the dual representation and the number of body sounds, which efficiently captured the self-attention fusion rule.

## 7. Challenges and Limitations

Our study also has limitations in data and model development. Beyond the number of body sounds combined, the varying duration of each instance can also influence the results. Here, we truncate recordings to 4 s, but a cough may last less than this, leaving only breathing sounds in the remaining time. A finer analysis taking this aspect into account should be considered in a follow-up study. Background noise presents another source of bias. Most recordings contain noise, which can potentially mislead the model. For instance, the model might predict a positive COVID-19 case solely due to the absence of background noise, as infected individuals are often isolated. To mitigate these biases, data collection should incorporate specific instructions. Participants could be instructed to record in quiet environments or produce a set number of coughs within a specific timeframe. Regarding model development, the joint representation brings marginal improvement over the model using only spectrogram features. This suggests that the contribution of waveform features is minimal compared to that of features derived from spectrograms to the model with joint representation. The choice of the wav2vec backbone model might not be optimal for the task at hand as it is pretrained on speech datasets, which differs from respiratory sounds such as cough and breath. A pre-trained model on a dataset comprising a multitude of respiratory sounds could potentially improve the effectiveness and generalizability of waveform features for respiratory diseases. In a future study, different waveform embeddings could be systematically compared. The same holds true for an extended analysis of different vision embedding backbones. Since the proposed FAIR framework is easily adaptable to such changes, we deem this a path for fruitful future research. While this study focused on COVID-19 detection based on the Coswara dataset, the FAIR approach can be generalized to combine various body sounds for identifying other respiratory illnesses. We plan to conduct further studies on other diseases where multi-instance and multi-modal features can be leveraged to enhance detection rates.

## 8. Conclusions

In this article, we study deep learning approaches to detect COVID-19 using body sounds. To this end, we propose FAIR, a multi-instance audio classification approach with attention-based fusion on waveform and spectrogram representation. We prove the effectiveness of our approach by conducting extensive experiments on the Coswara dataset. The results demonstrate that the fusion of body sounds using self-attention helps extract richer features that are useful for the classification of COVID-19-negative and -positive patients. In addition, we perform an in-depth analysis of the influence of the fusion rule on the performance. We find that the scaling property of self-attention shows great efficiency when more instances of body sounds and representations are adopted. The best setting with a combination of cough, breath, and speech sounds in waveform and spectrogram representation results in an AUC score of 0.8658, a sensitivity of 0.8057, and a specificity of 0.7958 on our test set. The sensitivity of our model exceeds 0.75, the required threshold of the COVID-19 screening test [10].

In addition, FAIR is not limited to COVID-19 detection. It can be adapted to other audio classification problems involving diverse combinations of multi-instance inputs. In our future work, we consider applying FAIR to other critical biomedical audio classification tasks. The framework can be extended in various ways, for example, by integrating multi-modal inputs, such as clinical lab values, with the spectrogram and waveform features derived from the audio signal. The attention-based fusion mechanism allows quantifying the feature attribution based on the attention weights. Particularly in the multi-modal setting, we propose carefully assessing the aforementioned attribution scores in order to derive further insights into the relevance of different audio or non-audio clinical biomarkers. Furthermore, as indicated in Truong et al. [36], simultaneously extracting and fusing multiple multi-modal embeddings could improve the overall model performance in classification tasks by leveraging complementary information within an extended feature space.

## Figures and Tables

**Table 1:** The statistics of audio length (in second) after the preprocessing step.

Body Sound	Min (s)	Max (s)	Median (s)	Mean (s)
Heavy cough	1.58	30.04	6.06	6.27
Deep breath	2.65	30.04	16.30	17.08
Normal counting	1.62	29.95	14.34	14.58
Fast counting	1.86	29.95	7.94	8.00
Phoneme /a/	1.19	29.95	10.03	10.53
Phoneme /e/	1.28	29.95	10.96	11.73
Phoneme /o/	1.37	29.95	10.41	11.19

**Table 2:** Baseline and benchmark experiments. The last experiment (BE3) is our proposed FAIR model that uses both waveform and spectrogram inputs and the body sound fusion unit.

No.	Representation	Architecture	Body Sound Fusion	No. Models
BA1	Waveform	wav2vec	None	7
BA2	Spectrogram	DeiT-S/16	None	7
BE1	Waveform	wav2vec	Attention	5
BE2	Spectrogram	DeiT-S/16	Attention	5
BE3	Spectrogram Waveform	DeiT-S/16 wav2vec	Attention	5

**Table 3:** Repartition of the subjects for the five-fold cross-validation scheme.

Subset	Label	Trial 1	Trial 2	Trial 3	Trial 4	Trial 5
Train	Negative	761	756	751	760	752
	Positive	146	151	155	146	154
Validation	Negative	184	189	194	185	193
	Positive	42	37	33	42	34

**Table 4:** Hyperparameter settings in baseline and benchmark experiments.

Architecture	wav2vec		DeiT-S/16		FAIR
Body sound fusion	None	Attention	None	Attention	Attention
Optimizer	AdamW	AdamW	AdamW	AdamW	AdamW
Base learning rate	$10^{-4}$	$10^{-4}$	$10^{-4}$	$10^{-4}$	$10^{-4}$
Weight decay	$10^{-3}$	$10^{-3}$	$10^{-1}$	$10^{-1}$	$10^{-3}$
Optimizer momentum	(0.9, 0.99)	(0.9, 0.99)	(0.9, 0.99)	(0.9, 0.99)	(0.9, 0.99)
Batch size	32	32	32	32	32
Training epochs	30	30	30	30	30
Learning rate scheduler	cosine	cosine	cosine	cosine	cosine
Warmup epochs	10	10	10	10	10
Loss function	BCE	BCE	BCE	BCE	BCE

**Table 5:** Baseline single representation, single body sound without fusion rules: five-fold mean and standard deviation of AUC scores related to baseline experiments utilizing only a single-input audio feature. A model with a DeiT-S/16 backbone relying only on an input spectrogram is benchmarked against a model with a wav2vec backbone relying only on a waveform input. The bold scores denote the highest performance achieved in this comparison.

Input Body Sound	wav2vec (BA1)	DeiT-S/16 (BA2)
Cough—heavy	0.4574 ± 0.0093	**0.7782 ± 0.0132**
Breath—deep	0.6597 ± 0.0222	**0.7552 ± 0.0254**
Counting—fast	0.7090 ± 0.0136	**0.7291 ± 0.0196**
Counting—normal	0.6285 ± 0.0155	**0.7943 ± 0.0326**
Phoneme /a/	0.6484 ± 0.0150	**0.7418 ± 0.0399**
Phoneme /e/	0.6209 ± 0.0197	**0.7399 ± 0.0318**
Phoneme /o/	0.5649 ± 0.0293	**0.7457 ± 0.0288**
Average	0.6127 ± 0.0751	**0.7549 ± 0.0215**

**Table 6:** Benchmark single and dual representation, multiple body sounds with fusion rules: five-fold mean and standard deviation of AUC related to benchmark experiments for fusing body sound instances and representations. The bold scores denote the highest performance achieved in this comparison. The *p*-value is calculated with a one-tailed Wilcoxon signed-rank test (*n* = 10) for each pair of experiments by repeating the five-fold cross-validation twice with different random seeds. The *p*-values for BE1 vs. BE2 and BE1 vs. BE3 are less than 0.001 and are omitted from the table.

		Model		*p*-Value
**Input Body Sounds**	**wav2vec (BE1)**	**DeiT-S/16 (BE2)**	**FAIR (BE3)**	**BE2 vs. BE3**
Speech	0.7562 ± 0.0152	0.8081 ± 0.0239	**0.8434 ± 0.0290**	< 0.001
Cough + Breath	0.6739 ± 0.0435	**0.7685 ± 0.0183**	0.7585 ± 0.0174	0.5000
Cough + Speech	0.7644 ± 0.0088	0.8315 ± 0.0306	**0.8584 ± 0.0308**	0.2460
Breath + Speech	0.7682 ± 0.0149	0.8122 ± 0.0125	**0.8319 ± 0.0187**	0.0137
Cough + Breath + Speech	0.7717 ± 0.0128	0.8241 ± 0.0266	**0.8658 ± 0.0115**	0.0019
Average	0.7469 ± 0.0369	0.8089 ± 0.0218	**0.8316 ± 0.0384**	

**Table 7:** Baseline dual representation, single body sound without fusion rules: five-fold mean and standard deviation of AUC scores related to baseline experiments utilizing concatenation of waveform and spectrogram representation with DeiT-S/16 and wav2vec backbones.

Input Body Sound	DeiT-S/16 & wav2vec
Cough—heavy	0.7426 ± 0.0268
Breath—deep	0.7661 ± 0.0113
Counting—fast	0.7698 ± 0.0204
Counting—normal	0.7581 ± 0.0938
Phoneme /a/	0.7577 ± 0.0213
Phoneme /e/	0.7299 ± 0.0174
Phoneme /o/	0.7394 ± 0.0168
Average	0.7519 ± 0.0137

**Figure A1:**
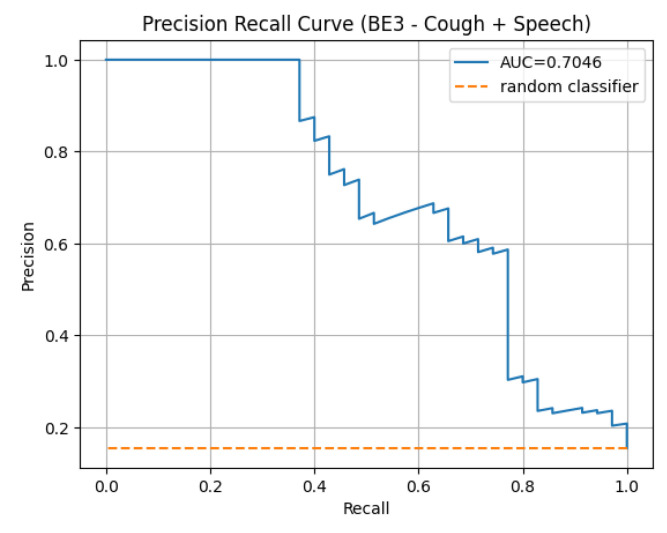
The precision–recall curve from one fold of the BE3 experiment using cough and speech sounds.

**Figure A2:**
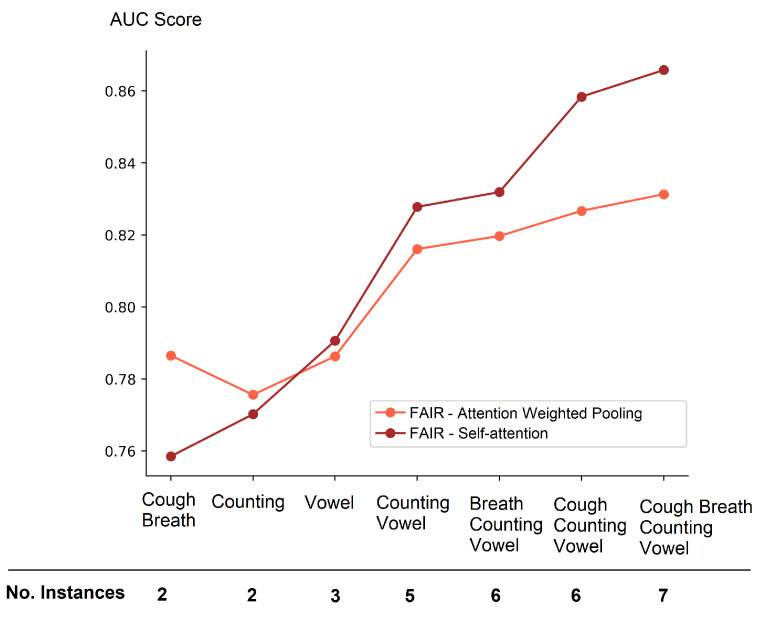
AUC scores of FAIR models sorted with respect to the number of instances in each combination. In addition to existing combinations, we added counting (two instances) and vowel (three instances) to comprehensively visualize the trend of AUC scores. While channel-weighted pooling appeared to saturate around 0.83, self-attention grew steadily without any sign of saturation.

## Data Availability

The datasets analyzed during the current study are published in the work of Bhattacharya et al. [42] and publicly available at https://github.com/iiscleap/Coswara-Data (accessed on 1 September 2021).

## Abbreviations

The following abbreviations are used in this manuscript:

MLP	Multilayer perceptron
ViT	Vision transformer
COVID-19	Coronavirus disease 2019
STFT	Short-time Fourier transform
SARS-CoV-2	Severe acute respiratory syndrome coronavirus 2
MFCC	Mel frequency cepstral coefficients
ZCR	Zero-crossing rate
KNN	K-nearest neighbors
SVM	Support vector machine
COPD	Chronic obstructive pulmonary disease
RNN	Recurrent neural network
LSTM	Long short-term memory
FAIR	Fused audio instance and representation
ROC	Receiver operating characteristic
AUC	Area under the ROC curve

## Appendix A. Additional Experimental Results

### Appendix A.1. Self-Attention Fusion with Only Waveform Inputs

**Table A1:** AUC, sensitivity, specificity, AUPRC, precision, F1, and accuracy of wav2vec models on different combination of body sounds using self-attention fusion.

Feature Extractor	wav2vec (BE1)						
**Body Sound**	**AUC**	**Sensitivity**	**Specificity**	**AUPRC**	**Precision**	**F1**	**Accuracy**
Speech	0.7562 ± 0.0152	0.3557 ± 0.0409	0.7592 ± 0.0586	0.3794 ± 0.0824	0.7028 ± 0.0689	0.4685 ± 0.0282	0.7504 ± 0.0460
Cough + Breath	0.6739 ± 0.0435	0.2694 ± 0.0363	0.7200 ± 0.0524	0.1583 ± 0.0181	0.7199 ± 0.0584	0.3904 ± 0.0453	0.6469 ± 0.0669
Cough + Speech	0.7644 ± 0.0088	0.3922 ± 0.0771	0.7906 ± 0.0937	0.4218 ± 0.0283	0.6628 ± 0.1056	0.4799 ± 0.0413	0.7708 ± 0.0729
Breath + Speech	0.7682 ± 0.0149	0.3747 ± 0.0675	0.6743 ± 0.0966	0.4526 ± 0.0167	0.6744± 0.1082	0.4705 ± 0.0343	0.7593 ± 0.0692
Cough + Breath + Speech	0.7717 ± 0.0128	0.3358 ± 0.0347	0.7236 ± 0.0669	0.3991 ± 0.0409	0.7460 ± 0.0757	0.4594 ± 0.0235	0.7265 ± 0.0514

### Appendix A.2. Self-Attention Fusion with Only Spectrogram Inputs

**Table A2:** AUC, sensitivity, specificity, AUPRC, precision, F1, and accuracy of DeiT-S/16 models on different combinations of body sounds using self-attention fusion. (*) Experiments on additional body sound combinations.

Feature Extractor	DeiT-S/16 (BE2)						
**Body sound**	**AUC**	**Sensitivity**	**Specificity**	**AUPRC**	**Precision**	**F1**	**Accuracy**
Speech	0.8081 ± 0.0239	0.7486 ± 0.0775	0.7717 ± 0.0711	0.5584 ± 0.0183	0.3865 ± 0.0598	0.5043 ± 0.0435	0.7681 ± 0.0570
Cough + Breath	0.7685 ± 0.0183	0.6400 ± 0.0642	0.8293 ± 0.0718	0.4749 ± 0.0680	0.4283 ± 0.0839	0.5043 ± 0.0443	0.8000 ± 0.0572
Cough + Speech	0.8315 ± 0.0306	0.7371 ± 0.0836	0.7927 ± 0.0892	0.5728 ± 0.0558	0.4229 ± 0.1148	0.5251 ± 0.0802	0.7841 ± 0.0756
Breath + Speech	0.8122 ± 0.0125	0.6571 ± 0.0313	0.8796 ± 0.0298	0.5879 ± 0.0615	0.5077 ± 0.0623	0.5699 ± 0.0339	0.8451 ± 0.0250
Cough + Breath + Speech	0.8241 ± 0.0266	0.6914 ± 0.0796	0.8408 ± 0.0838	0.6159 ± 0.0174	0.4741 ± 0.1064	0.5502 ± 0.0658	0.8177 ± 0.0696
Counting (fast + normal) (*)	0.7467 ± 0.0124	0.6629 ± 0.0946	0.7790 ± 0.0774	0.4456 ± 0.0368	0.2611 ± 0.0603	0.3860 ± 0.0553	0.5849 ± 0.1302
Phoneme (/a/-/e/-/o/) (*)	0.7806 ± 0.0208	0.7886 ± 0.0100	0.6827 ± 0.0753	0.4311 ± 0.0258	0.2705 ± 0.0637	0.3956 ± 0.0681	0.5752 ± 0.2151

**Table A3:** AUC, sensitivity, and specificity of ResNet50 models on different combination of body sounds using self-attention fusion.

Feature Extractor	ResNet50		
**Body Sound**	**AUC**	**Sensitivity**	**Specificity**
Speech	0.7531 ± 0.0362	0.7314 ± 0.0983	0.6817 ± 0.0818
Cough + Breath	0.7585 ± 0.0259	0.6400 ± 0.0859	0.8188 ± 0.0832
Cough + Speech	0.7817 ± 0.0282	0.8000 ± 0.1352	0.6628 ± 0.0992
Breath + Speech	0.7862 ± 0.0238	0.7314 ± 0.0878	0.7466 ± 0.1058
Cough + Breath + Speech	0.8026 ± 0.0229	0.6914 ± 0.1120	0.7959 ± 0.1175

### Appendix A.3. FAIR

**Table A4:** AUC, sensitivity, specificity, AUPRC, precision, F1 and accuracy of FAIR models (DeiT-S/16 & wav2vec) on different combination of body sounds. (*) Experiments on additional body sound combinations.

Feature Extractors	DeiT-S/16 & wav2vec (BE3)						
**Body Sound**	**AUC**	**Sensitivity**	**Specificity**	**AUPRC**	**Precision**	**F1**	**Accuracy**
Speech	0.8434 ± 0.0290	0.7429 ± 0.0767	0.8356 ± 0.0266	0.5566 ± 0.0371	0.4551 ± 0.0235	0.5619 ± 0.0234	0.8212 ± 0.0146
Cough + Breath	0.7585 ± 0.0174	0.6629 ± 0.0874	0.8168 ± 0.0754	0.4971 ± 0.0698	0.4199 ± 0.0806	0.5030 ± 0.0370	0.7222 ± 0.1352
Cough + Speech	0.8584 ± 0.0308	0.8171 ± 0.1063	0.7738 ± 0.0977	0.6016 ± 0.0648	0.4205 ± 0.0836	0.5447 ± 0.0588	0.7805 ± 0.0768
Breath + Speech	0.8319 ± 0.0187	0.7771 ± 0.0554	0.7895 ± 0.0644	0.6330 ± 0.0529	0.4164± 0.0690	0.5365 ± 0.5455	0.7876 ± 0.5455
Cough + Breath + Speech	0.8658 ± 0.0115	0.8057 ± 0.0554	0.7958 ± 0.0678	0.6383 ± 0.0255	0.4352 ± 0.0796	0.5584 ± 0.0506	0.7974 ± 0.0546
Counting (fast + normal) (*)	0.7702 ± 0.0313	0.7086 ± 0.0836	0.7717 ± 0.0470	0.5009 ± 0.0347	0.2851 ± 0.0626	0.4000 ± 0.0549	0.6221 ± 0.1796
Phoneme (/a/-/e/-/o/) (*)	0.7906 ± 0.0095	0.7886 ± 0.0530	0.6848 ± 0.0499	0.4743 ± 0.0417	0.3544 ± 0.1242	0.4429 ± 0.0567	0.6805 ± 0.1248

### Appendix A.4. Remarks Regarding the AUPRC

As the classes are imbalanced in our study, we provided the AUPRC (Area Under the Precision-Recall Curve). The performance of our models can be compared with the area under the curve of a random classifier. The random classifier in our study is defined as a horizontal line y=35/226≈0.15, which is the ratio of the positive samples to the total samples. Figure A1 shows an example of the Precision-Recall Curve of our experiment BE3 using cough and speech sounds.

## Appendix B. Ablation Study

### Appendix B.1. Feature Extractors

In addition to feature extractors that use self-attention layers, i.e., ViT and wav2vec, we train two CNN-based feature extractors for waveform and spectrogram inputs. We employ a ResNet50 [44] for spectrogram and simple 1D-CNN with four convolutional blocks, denoted as 1D-CNN4, for waveform. Our setup is similar to the BA1-2 and BE1-2 experiments, only replacing DeiT-S/16 with ResNet50 and wav2vec with 1D-CNN network. The AUC, sensitivity and specificity are shown in Table A5 and Table A6. Both ResNet50 and DeiT-S/16 are pretrained on the ImageNet classification task, where DeiT-S/16 already outperforms ResNet50. When we transfer both pretrained models to our task, the results demonstrate that DeiT-S/16 also outperforms ResNet50 consistently in all experiments. This is expected because the pretrained DeiT-S/16 is initially more powerful than ResNet50, which is observed in experiments on natural images. In addition, 1D-CNN4 performs better when only a single body sound is used but less than wav2vec when applying body sound fusion rules. Because our objective is to leverage the combination of body sounds, the wav2vec net is preferred based on its performance.

**Table A5:** AUC, sensitivity, and specificity of ResNet50 and 1D-CNN4 as feature extractors on a single body sound.

Feature Extractor	1D-CNN4			ResNet50		
**Body Sound**	**AUC**	**Sensitivity**	**Specificity**	**AUC**	**Sensitivity**	**Specificity**
Cough-heavy	0.6396 ± 0.0839	0.8800 ± 0.0662	0.4042 ± 0.0555	0.6855 ± 0.0607	0.6571 ± 0.0767	0.7025 ± 0.0531
Breath-deep	0.6559 ± 0.0355	0.5886 ± 0.0690	0.7194 ± 0.1241	0.7387 ± 0.0244	0.5829 ± 0.0878	0.8545 ± 0.0561
Counting-fast	0.7162 ± 0.0400	0.5943 ± 0.1134	0.7885 ± 0.0829	0.7162 ± 0.0400	0.5943 ± 0.1134	0.7885 ± 0.0829
Counting-normal	0.6519 ± 0.0071	0.5143 ± 0.0866	0.7665 ± 0.0957	0.7082 ± 0.0395	0.5371 ± 0.0911	0.8188 ± 0.0570
Phoneme /a/	0.7014 ± 0.0387	0.6057 ± 0.1321	0.7560 ± 0.1331	0.7014 ± 0.0387	0.6057 ± 0.1321	0.7560 ± 0.1331
Phoneme /e/	0.6588 ± 0.0627	0.6571 ± 0.1743	0.6461 ± 0.1314	0.6588 ± 0.0627	0.6571 ± 0.1743	0.6461 ± 0.1314
Phoneme /o/	0.6327 ± 0.0145	0.6400 ± 0.0736	0.6345 ± 0.0880	0.7004 ± 0.0785	0.8057 ± 0.0874	0.5780 ± 0.1591

### Appendix B.2. Fusion Unit

In addition to self-attention, we perform an ablation study on weighted attention pooling, a different attention-based fusion technique. In attention-weighted pooling, we compute the joint feature vector z to be the weighted sum of body sound features:

(A1) $$z=\sum_{j=1}^{c}w_{j}f_{j}$$

where $w_{j}$ is the attention weight given to feature $f_{j}$. To obtain $w_{j}$, we follow the idea of the squeeze-and-excitation block [45]. Table A7 shows that the AUC scores of attention-weighted pooling remain almost unchanged. The best AUC score in FAIR with attention-weighted pooling is 0.8313, which is roughly the same as the best one produced by DeiT-S/16, 0.8367 (Table A2). In contrast, self-attention produced a steady rise in AUC scores, with the best one being 0.8658, a 0.03 increase from using only DeiT-S/16. Figure A2 shows the trend of AUC scores as more instances are added. Even AUC scores rise proportionally to the number of body sounds. Fusion with attention-weighted pooling reaches saturation at 0.83, while self-attention trends improve steadily.

**Table A6:** AUC, sensitivity, and specificity of ResNet50 and 1D-CNN4 as feature extractors on different combinations of body sounds using self-attention fusion.

Feature Extractor	1D-CNN4			ResNet50		
**Body Sound**	**AUC**	**Sensitivity**	**Specificity**	**AUC**	**Sensitivity**	**Specificity**
Speech	0.7235 ± 0.0052	0.5543 ± 0.0464	0.8335 ± 0.0399	0.7531 ± 0.0362	0.7314 ± 0.0983	0.6817 ± 0.0818
Cough + Breath	0.6900 ± 0.0145	0.7886 ± 0.1273	0.5278 ± 0.1150	0.7585 ± 0.0259	0.6400 ± 0.0859	0.8188 ± 0.0832
Cough + Speech	0.7362 ± 0.0081	0.6229 ± 0.0732	0.7770 ± 0.0651	0.7817 ± 0.0282	0.8000 ± 0.1352	0.6628 ± 0.0992
Breath + Speech	0.7351 ± 0.0060	0.5943 ± 0.0214	0.8492 ± 0.0332	0.7862 ± 0.0238	0.7314 ± 0.0878	0.7466 ± 0.1058
Cough + Breath + Speech	0.7596 ± 0.0122	0.6914 ± 0.0709	0.7539 ± 0.0668	0.8026 ± 0.0229	0.6914 ± 0.1120	0.7959 ± 0.1175

**Table A7:** AUC, sensitivity, and specificity of FAIR4Cov (DeiT-S/16 and wav2vec) on different combinations of body sounds using attention-weighted pooling. (*) Experiments on additional body sound combinations.

Feature Extractors	DeiT-S/16 & wav2vec					
**Fusion Rules**	**Attention-Weighted Pooling**			**Self-Attention**		
**Body Sound**	**AUC**	**Sensitivity**	**Specificity**	**AUC**	**Sensitivity**	**Specificity**
Speech	0.8161 ± 0.0238	0.8172 ± 0.1166	0.6932 ± 0.1125	0.8434 ± 0.0290	0.7429 ± 0.0767	0.8356 ± 0.0266
Cough + Breath	0.7865 ± 0.0173	0.6514 ± 0.0911	0.8544 ± 0.0673	0.7585 ± 0.0174	0.6629 ± 0.0874	0.8168 ± 0.0754
Cough + Speech	0.8267 ± 0.0102	0.8628 ± 0.0457	0.6911 ± 0.0525	0.8584 ± 0.0308	0.8171 ± 0.1063	0.7738 ± 0.0977
Breath + Speech	0.8197 ± 0.0317	0.7257 ± 0.0690	0.8168 ± 0.0638	0.8319 ± 0.0187	0.7771 ± 0.0554	0.7895 ± 0.0644
Cough + Breath + Speech	0.8313 ± 0.0176	0.6743 ± 0.0428	0.0867 ± 0.0278	0.8658 ± 0.0115	0.8057 ± 0.0554	0.7958 ± 0.0678
Counting (fast + normal) (*)	0.7756 ± 0.0434	0.7086 ± 0.0911	0.7833 ± 0.0512	0.7702 ± 0.0313	0.7086 ± 0.0836	0.7717 ± 0.0470
Vowel (a, e, o) (*)	0.7863 ± 0.0211	0.7486 ± 0.0754	0.7068 ± 0.0797	0.7906 ± 0.0095	0.7886 ± 0.0530	0.6848 ± 0.0499

## Appendix C. Model Complexity

Below, we report the number of trainable parameters for our FAIR approach with different combinations of respiratory sounds. As the feature extractors are frozen during the training, the trainable parameters come from the fine-tuning unit and the classifier.

**Table A8:** Number of trainable parameters in the FAIR approach for different combinations of body sounds.

Input Body Sounds	Number of Trainable Parameters
Speech	22.07M
Cough + Breath	21.97 M
Cough + Speech	22.10 M
Breath + Speech	22.10 M
Cough + Breath + Speech	22.14 M
